# Intermediate Hosts of *Angiostrongylus cantonensis* in Tenerife, Spain

**DOI:** 10.1371/journal.pone.0120686

**Published:** 2015-03-24

**Authors:** Aarón Martin-Alonso, Estefanía Abreu-Yanes, Carlos Feliu, Santiago Mas-Coma, María Dolores Bargues, Basilio Valladares, Pilar Foronda

**Affiliations:** 1 Instituto Universitario de Enfermedades Tropicales y Salud Pública de Canarias, Universidad de la Laguna, La Laguna, Islas Canarias, España; 2 Departamento de Microbiología y Parasitología, Universidad de Barcelona, Barcelona, Cataluña, España; 3 Departamento de Parasitología, Facultad de Farmacia, Universidad de Valencia, Burjassot, Spain; Royal Tropical Institute, NETHERLANDS

## Abstract

The nematode *Angiostrongylus cantonensis* is the causative agent of human angiostrongyliasis, the main clinical manifestation of which is eosinophilic meningitis. Although this parasite has been found recently in its definitive rat host in Tenerife (Canary Islands, Spain), showing a widespread distribution over the north-east part of the island, there are no available data regarding which snail and/or slug species are acting as intermediate hosts on this island. Consequently, the objective of this work was to determine the possible role of three mollusc species, *Plutonia lamarckii*, *Cornu aspersum* and *Theba pisana*, as intermediate hosts of *A*. *cantonensis* in Tenerife. Between 2011 and 2014, 233 molluscs were collected from five biotopes where rats had been found previously to harbor either adult worms or antibodies against *A*. *cantonensis*, and the identification was carried out on the basis of morphological features and a LAMP technique. The prevalence of *A*. *cantonensis* larvae in the mollusc samples, based on morphological identification, was 19.3%, whereas 59 out of the 98 individuals (60.2%) analyzed by LAMP were positive. Positive results were obtained for the three mollusc species analyzed and two of the positive samples, both obtained from *P*. *lamarckii*, were confirmed as positive by 18S rRNA and ITS1 PCR. Sequence analysis of 18S rRNA PCR products showed 100% similarity with previously published *A*. *cantonensis* sequences. These results may be relevant from a public health point of view, since all the biotopes from which the samples were obtained were in inhabited areas or areas with human activity, but it is also important from the perspective of a possible transmission to other accidental hosts, such as dogs and horses, animals that are present in some of the areas analyzed.

## Introduction

The nematode *Angiostrongylus cantonensis* is the causative agent of human angiostrongyliasis, which in its severe form is characterized by eosinophilic meningitis (or meningoencephalitis), with marked cerebrospinal fluid (CSF) eosinophilia [1]. The life cycle of this nematode involves rats and molluscs as definitive and intermediate hosts, respectively, whereas humans are accidental hosts infected through the consumption of raw or undercooked molluscs that contain the infective third stage larvae (L3) [2]. Once the nematode has been ingested by a person, it can reach the central nervous system or, less frequently, the eye, causing eosinophilic meningitis or ocular angiostrongyliasis, respectively [3]. Human angiostrongyliasis presents a broad clinical spectrum, from a mild disease to a form of eosinophilic meningitis or, uncommonly, encephalitis [4]. As a result, neurological damage and even death may occur, especially if prompt and proper treatment is not administered [5–7].

From its original range in southeastern China, *A*. *cantonensis* spread throughout many tropical and sub-tropical regions of the world during the 20th century [8,9]. This rapid geographical spread coincided with globalization and has probably been facilitated by the unintentional transport of infected hosts in ships and planes [10]. Once introduced into a new area, the nematode may easily establish itself in the local fauna since a large number of mollusc species can act as intermediate hosts and rats are ubiquitous [8].

Detection of *A*. *cantonensis* L3s in snails by digestion of snail tissues, followed by microscopic examination, is time-consuming and requires expertise in identifying *A*. *cantonensis* larvae [11]. Molecular detection using the polymerase chain reaction (PCR) has been used to circumvent these problems associated with morphological identification of *Angiostrongylus* worms. Genomic DNA suitable for PCR detection can be extracted from various types of material, including intact worms in all developmental stages, tissues from intermediate, definitive and paratenic hosts, and rat droppings [11]. A loop-mediated isothermal DNA amplification (LAMP) technique has also been used to detect *A*. *cantonensis* in invasive snail species [12,13] and presents several advantages when compared to PCR. For instance, the LAMP technique does not require special equipment; amplification of the target DNA can be performed in a water bath or heat block and the end-point analysis can be achieved by visual inspection directly in the reaction tube. The LAMP assay has already been applied to the detection and identification of other parasites of humans and animals [13].

*A*. *cantonensis* has been detected recently in black rats (*Rattus rattus*) in the Canary Islands [14], an archipelago very rich in gastropod species, among which many are endemic [15]. To our knowledge, this is the only location in Europe where the parasite has been detected. Nevertheless, there are no data regarding which snail and/or slug species are acting as intermediate hosts in the Canary Islands. Hence, the possible sources of human infection have not been identified until now. The aim of this study was to determine the possible role of three mollusc species, *Plutonia lamarckii*, *Cornu aspersum* and *Theba pisana*, as intermediate hosts of *A*. *cantonensis* in Tenerife.

## Materials and Methods

### Sampling areas

**Fig 1:**
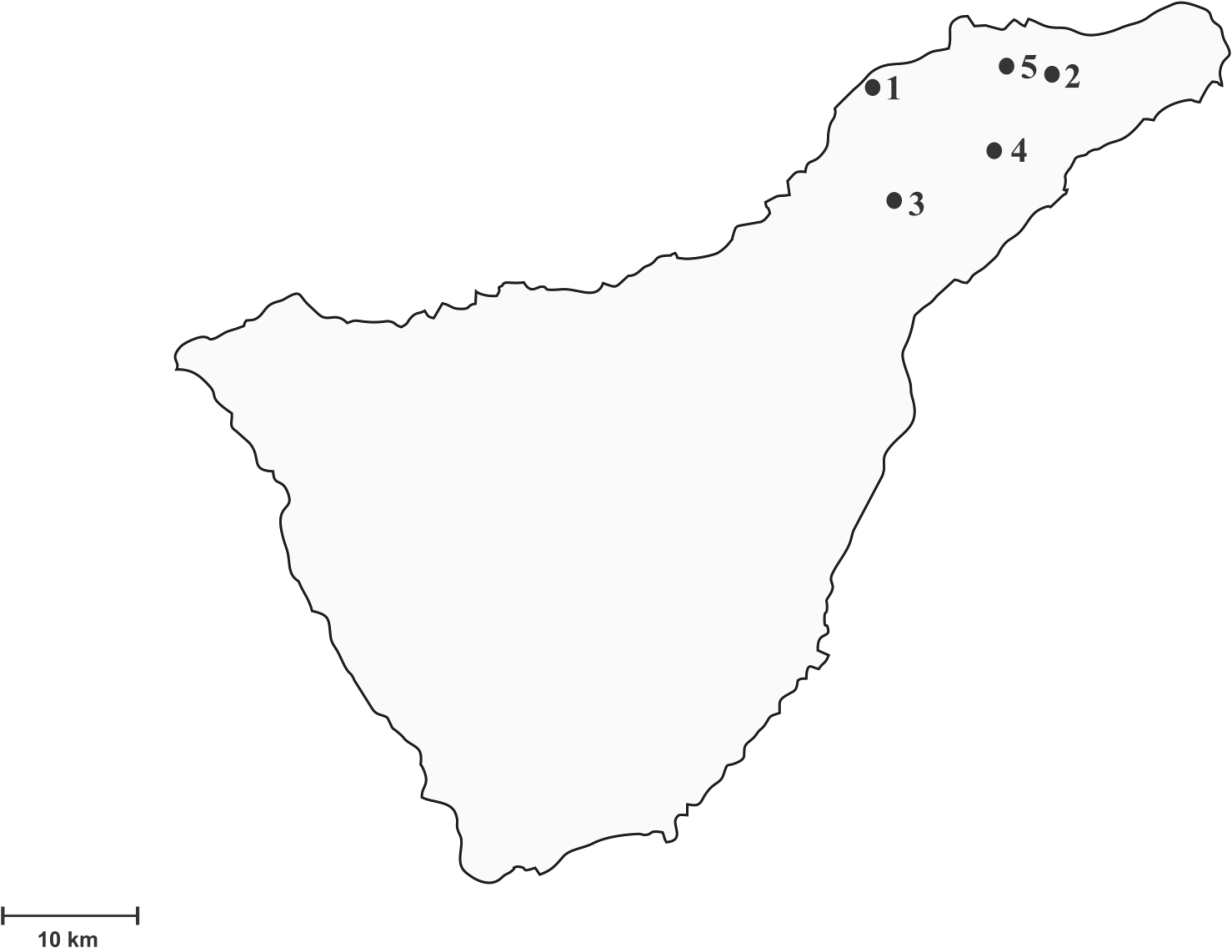
Geographical distribution of the sampling areas in Tenerife. 1, El Pris; 2, Pico del Inglés; 3, La Esperanza; 4, La Laguna; 5, Pedro Álvarez.

**Fig 2:**
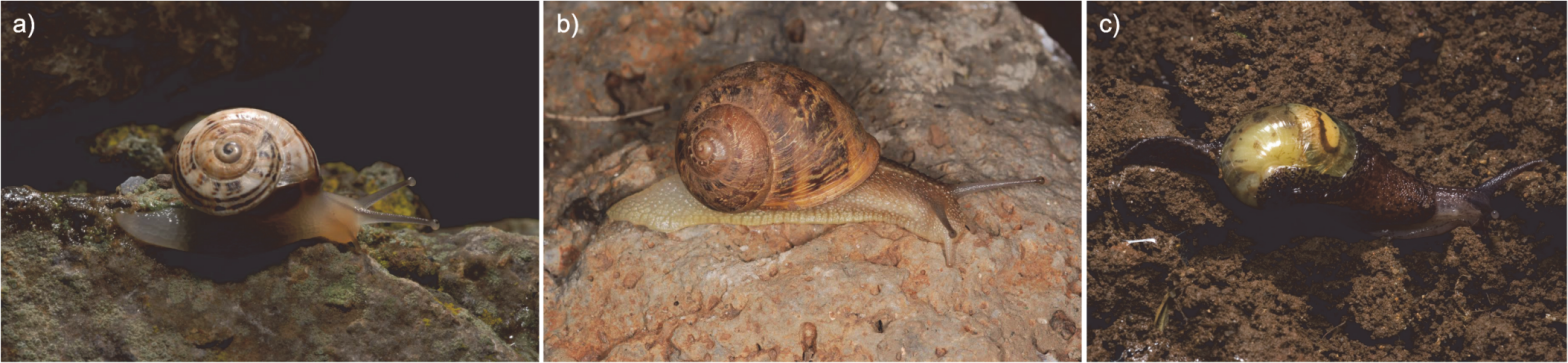
Mollusc species included in the study. *Theba pisana* (a), *Cornu aspersum* (b), *Plutonia lamarckii* (c).

Between 2011 and 2014, 233 molluscs were collected at several locations in Tenerife. This island, with an area of 2068 km^2^, is the largest of the Canary Islands and of the Macaronesian region as a whole. The biotopes sampled in this study included three laurel forest areas (Pedro Álvarez, La Esperanza and Pico del Inglés) and two inhabited areas close to urban centers (La Laguna and El Pris) (Fig. 1). The mollusc species analyzed were the endemic semi-slug *P*. *lamarckii* and the snails *T*. *pisana* and *C*. *aspersum* (Fig. 2). Either adult worms of *A*. *cantonensis* or black rats carrying antibodies against *A*. *cantonensis* had been previously found in all the five locations [2].

### Ethical statement

This field study did not involve endangered or protected species. Animal trapping in protected areas (Pedro Álvarez and Pico del Inglés) was approved by the Área de Medio Ambiente del “Excmo. Cabildo Insular” de Tenerife.

### Morphological identification of *Angiostrongylus cantonensis* larvae

Tissue samples were cut from the posterior end of the mollusc's foot and placed in 1 ml of 0.01% pepsin-0.7% HCl in individual wells of a 24-well culture dish for digestion of the tissue. This method was combined with direct observation of mollusc tissues after mincing. Taxonomic identification of nematodes was based on morphological characters and morphometric parameters [16].

### DNA extraction

Other individuals of the same species were used for molecular detection of *A*. *cantonensis* larvae. Tissue samples were cut from the posterior end of the mollusc’s foot and DNA was extracted by digestion with 4 mg proteinase K/ml in a buffer consisting of 30 mM Tris-HCl (pH 8), 0.4% SDS, and 10 mM EDTA overnight at 56°C, followed by incubation at 95°C for 10 min to inactivate the proteinase K. After having inactivated the proteinase K, DNA extraction continued by following the method used by López et al. [17].

### LAMP method for the detection of *A*. *cantonensis* in mollusc tissue

**Fig 3:**
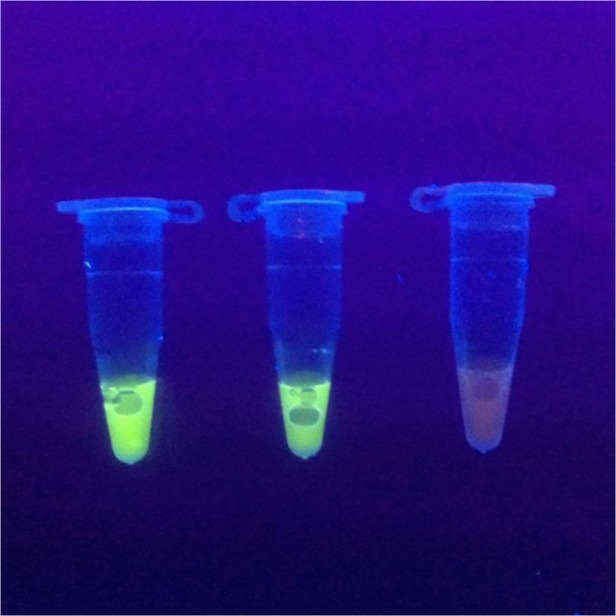
Detection of *Angiostrongylus cantonensis* by LAMP reaction. tube 1, DNA isolated from *Plutonia lamarckii* tissue; tube 2, DNA obtained from *A*. *cantonensis* adult worms; tube 3, negative control.

The LAMP assay was performed according to [13]. Briefly, the assay was carried out in a 25 μl reaction system containing 10x Bst-DNA polymerase buffer (3 μl), deoxynucleotide triphosphates (0.5 mM for each), MgSO4 (2 mM), a forward inner primer (FIP) (5′- CTCATCATCAACCACCCACCCCTAGCATCATCTACGTCGTC-3′) and a backward inner primer (BIP) (5′-AGAAACCACCAACACATATACACGTATACCACCAACTTTAGCGA-3′) (1.6 μM for each), loop-F (5′- GGGTGGTGATGTAGTAGCTA-3′) and loop-B (5′- TCACCTAGTGTATGATGGT-3′) (0.8 μM for each), 0.4 μM of outer primers F3(5′- CCACCACAAAACACAAACA-3′) and B3 (5′-GTGTTGAGCTCTAACGGT-3′), Bst DNA polymerase (8 U) (New England BioLabs) and DNA template (1 μl, approximately 30 ng). A reaction system with no DNA template was used as the negative control. The mixtures were incubated at 65°C for 45 min, and then heated at 80°C for 10 min to terminate the reaction. LAMP amplification results were visually detected under UV light after adding 2 μl of 20x EvaGreen I (Biotium) to the reaction tubes. Solutions turned green in the case of positive LAMP amplification; otherwise, they remained orange (Fig. 3).

### PCR amplification

DNA extracted from LAMP-positive tissue samples was amplified by conventional PCR targeting the 18S rRNA [18]. Briefly, the 50 μl PCR mixtures contained 0.4 μM of each primer (Angio-F and Angio-R) and 1.25 U of BioTaq DNA pol (Bioline). Amplification was carried out using the following cycling parameters: 95°C for 5 min, 45 cycles of 95°C for 15 s, 65°C for 15 s, and 72°C for 1 min, and 72°C for 10 min.

18S rRNA positive samples were confirmed to be infected with *A*. *cantonensis* by PCR targeting the first internal transcribed spacer (ITS1), which is comparatively more variable than the rRNA coding regions and therefore allows the identification of closely related species [19]. The ITS1 PCR was adapted from a real-time PCR assay [19], and it was conducted in a 50 μL reaction mixture containing 200 μM of each dNTP (Bioline), 1.5 mM MgCl_2_ (Bioline) 0.4 μM of each primer (AcanITS1F1 and AcanITS1R1) and 1 U of BioTaq DNA pol (Bioline). Amplification was performed using the following cycling parameters: 94°C for 2 min, 35 cycles of 94°C for 30 s, 62°C for 30 s, and 72°C for 30 s, and 72°C for 7 min.

### Statistical analysis

Binary logistic regression was carried out to analyze the influence of the covariables (mollusc species and location) on the presence or absence of *A*. *cantonensis* (outcome variable), determined by LAMP. Odds ratios (ORs) and 95% confidence intervals (CI) were calculated for these associations. Interactions between covariables within the logistic model were also tested by adding an interaction variable for mollusc species and location. A probability value less than < 0.05 was considered as statistically significant. 95% CI for prevalence obtained with the LAMP technique were calculated.

## Results

**Fig 4:**
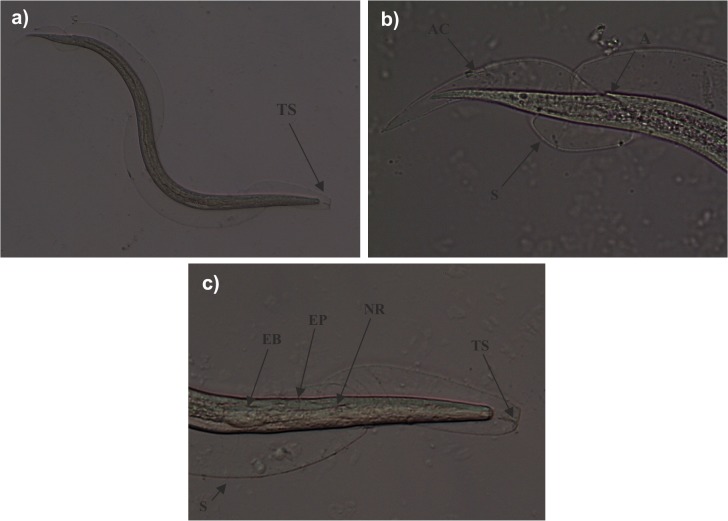
Light microscopy of third stage larvae (L3) morphologically compatible with *Angiostrongylus cantonensis*, obtained from *Theba pisana*. General view of third-stage larvae with one sheath (*S*). A characteristic “T”-shaped structure (*TS*) is apparent at the anterior end of the sheath that surrounds the L3 larva (a). Posterior end of L3 larva with anus (*A*) at subterminal position. Anus cuticle (*AC*) is completely molted and can be seen on the sheath (b). Anterior end of L3 larva with esophagus bulbus (*EB*), excretory pore (*EP*) and nervous ring (*NR*) (c).

*A*. *cantonensis* larvae were present in 19.3% of the molluscs analyzed morphologically but in 60.2% (59/98) of those analyzed by LAMP (Table 1). Morphological features of third-stage larvae are illustrated in Fig. 4. All three species showed positive LAMP results, whereas *A*. *cantonensis* was detected by the morphological method only in *T*. *pisana* and *P*. *lamarckii*, although the sample size of *C*. *aspersum* specimens was limited. With regard to the results obtained by LAMP technique, the binary logistic regression showed that only the covariable mollusc species was significantly associated with the presence of *A*. *cantonensis* (*P*<0.01). More concretely, *P*. *lamarckii* specimens showed a more than 6-fold increased odd of being infected with *A*. *cantonensis*, when compared with *T*. *pisana* specimens (OR = 6.9, 95% CI 1.99–23.87, *P*<0.01). On the other hand, no significant interaction was found between the two covariables, mollusc species and location.

**Table 1:** Percentage prevalence of *Angiostrongylus cantonensis* larvae in molluscs and numbers of molluscs testing positive in samples from Tenerife, based on morphological (P) and molecular identification (P*).

Area of study	Mollusc species	P (%) (+/n)	P* (%) (+/n) [95% CI]
**El Pris**			
	*Theba pisana*	53.1 (17/32)	-
	*Plutonia lamarckii*	-	-
	*Cornu aspersum*	0 (0/1)	-
	Total	51.5 (17/33)	-
**Pico del Inglés**			
	*Theba pisana*	33.3 (1/3)	-
	*Plutonia lamarckii*	0 (0/32)	77.8 (7/9) [45.3–93.7]
	*Cornu aspersum*	0 (0/5)	-
	Total	2.5 (1/40)	77.8 (7/9) [45.3–93.7]
**La Laguna**			
	*Theba pisana*	21.9 (25/114)	35 (7/20) [18.1–56.7]
	*Plutonia lamarckii*	-	-
	*Cornu aspersum*	0 (0/3)	50 (9/18) [29–71]
	Total	21.3 (25/117)	42.1 (16/38) [27.9–57.8]
**La Esperanza**			
	*Theba pisana*	0 (0/18)	-
	*Plutonia lamarckii*	0 (0/5)	55.6 (10/18) [33.7–75.4]
	*Cornu aspersum*	-	-
	Total	0 (0/23)	55.6 (10/18) [33.7–75.4]
**Pedro Álvarez**			
	*Theba pisana*	-	-
	*Plutonia lamarckii*	10 (2/20)	78.8 (26/33) [62.2–89.3]
	*Cornu aspersum*	-	-
	Total	10 (2/20)	78.8 (26/33) [62.2–89.3]
**Total**		19.3 (45/233)	60.2 (59/98) [50.5–69.8]

*n., number of molluscs studied; +, number of positive samples; P, prevalence; CI, confidence interval.

Two of the samples that tested positive with LAMP were confirmed as positive by 18S rRNA PCR (GenBank Accession number KM096415). Both were from *P*. *lamarckii* specimens. Sequences exhibited 100% similarity to an 18S rRNA gene partial sequence of *A*. *cantonensis* retrieved from GenBank (AY295804.1) [18] and to a sequence obtained from *A*. *cantonensis* third stage larvae of *Parmarion* cf. *martensi* [20]. Both samples were confirmed to be infected with *A*. *cantonensis* by amplification of the ITS1 region.

## Discussion

*A*. *cantonensis* was detected by morphological and/or molecular methods in all three mollusc species and from all the areas studied. The three species are common and widespread over the north-east part of Tenerife.

This finding is in concordance with the previously demonstrated capacity of *A*. *cantonensis* to infect naturally many diverse freshwater and terrestrial mollusc species [8]. The prevalence recorded in this study via LAMP (60.2%) is higher than that found in other studies: 9% via real-time PCR in *Pomacea maculata* [21]; 14–31% by morphological identification in *Pomacea canaliculata* [22]; and 25% and 6.5% by morphological identification in *Achatina fulica* and *P*. *canaliculata*, respectively [23]. These differences could be due in part to a higher sensitivity of LAMP compared to the other methods. On the other hand, the prevalence observed in this work was lower than those found in *P*. *martensi* by PCR (73.5% and 74.1%) [19,20].

There is a huge variability of mollusc species in the Canary Islands [15], and we only analyzed three mollusc species in this study. Therefore, the rest of mollusc species should be studied to assess the real range of mollusc species that are acting as intermediate hosts of *A*. *cantonensis* in this archipelago. To our knowledge, this work constitutes the first finding of *A*. *cantonensis* in the mollusc species *T*. *pisana* and *P*. *lamarckii*. The genus *Plutonia* belongs to the family Vitrinidae, which contains mainly Palaearctic semislugs [24]. This genus has colonized the Macaronesian Islands (Azores, Madeira, Canary Islands and Cape Verde) [24], with six endemic species, including *P*. *lamarckii*, now occurring in the Canary Islands. Whereas the highest diversity of European vitrinids can be found above 1000 m altitude, their highest diversity in the Canary Islands is below 500 m [24], corresponding to the most inhabited zones of these islands. The possible role as intermediate hosts of *A*. *cantonensis* of the other vitrinid species in Tenerife should be assessed. Nevertheless, *P*. *lamarckii* is mainly distributed in laurel forest and rarely occurs in the inhabited areas sampled in this study. Thus, this study suggests that *T*. *pisana* and *C*. *aspersum* may be important species implicated in the transmission of the parasite to its definitive and accidental hosts, including humans, in inhabited areas such as La Laguna and El Pris, whereas *P*. *lamarckii* might be involved in this transmission in laurel forest areas. Supporting this hypothesis, introduced rats in Tenerife are known to prey on endemic mollusc species, such as *Plutonia* spp. [25], a phenomenon that could explain the notable percentage of rats (55.6%) carrying antibodies against *A*. *cantonensis* in a previous study carried out mainly in rural areas of Tenerife [2], along with the high prevalence of *P*. *lamarckii* specimens carrying *A*. *cantonensis* larvae in this study (71.67% by LAMP technique). With regard to *C*. *aspersum*, this snail species has been reported as an unsuitable host [26], although it has been used recently to transmit infection to rats experimentally [27]. This snail species is currently cooked for food in the Canary Islands, where several snail gardens have been created to produce them. In accordance with the introduced *T*. *pisana*, it is native to coastal regions of the Mediterranean and Western Europe, as far north as Ireland and Wales [28], whereas *C*. *aspersum* is also prevalent in Western Europe, along the Atlantic coast and at the Balkan. Because of its gastronomic use this species also occurs in Africa, Oceania, America and Australia [29].

Molecular methods to detect *A*. *cantonensis* were used in this study because morphological identification based on pepsin digestion can only identify the larvae to the superfamily level [19]. In this study, we found that only 3.4% of the molluscs testing positive via LAMP were confirmed by PCR, possibly because LAMP technique is able to detect DNA quantities as low as 0.001 ρg of *A*. *cantonensis* genomic DNA [12,13]. Therefore, LAMP technique can demonstrate the presence of *A*. *cantonensis* genomic DNA in lightly-infected molluscs. It should be also taken into account that LAMP is hardly inhibited by impurities such as those found in tissue-derived DNA samples [30]. LAMP shows not only high sensitivity but also exhibits high specificity, since several authors have demonstrated the absence of cross-reactions by using DNA samples obtained from various other parasites [13].

Our findings may be important from a public health point of view, since all the biotopes analyzed were in inhabited areas (La Laguna and el Pris) near to urban centers or other places frequented by people for leisure (Pedro Álvarez, Pico del Inglés and La Esperanza). There is also the possibility of infecting other mammal species in Tenerife as both dogs and horses, since these animals have been reported as accidental hosts [31]. Natural infections of horses may produce verminous encephalomyelitis with tetraparesis as the principal clinical features. Canine neural angiostrongyliasis caused by this nematode has been reported in Australia and elsewhere [32].

Although raw snails and slugs are not part of the Canary Islands diet, there is a risk associated with ingestion of undercooked *C*. *aspers*um or accidentally ingesting snails or slugs in vegetable produce, and a slight risk of transmission to humans from contact with the mucus deposited by them [33]. The zoonotic relevance of this study is underlined by the finding of the first case of a human patient carrying antibodies against *A*. *cantonensis* in Tenerife (Martin-Alonso pers. comm.), indicating that transmission to humans may be taking place at present. The historical absence of the parasite in the Canary Islands may be an artefact of the lack of familiarity with this nematode and the diseases that it causes among physicians, which is reflected in the fact that human angiostrongyliasis is not currently included in the differential diagnosis of patients with meningitis in the Canary Islands. Consequently, further investigations to ascertain the real incidence of human angiostrongyliasis in the Canary Islands are required, in order to assess the role of this nematode as a cause of eosinophilic meningitis in the archipelago.
